# 5-Carb­oxy-2,4-dihy­droxy­anilinium chloride

**DOI:** 10.1107/S1600536810033337

**Published:** 2010-08-21

**Authors:** Syeda Sohaila Naz, Nazar Ul Islam, M. Nawaz Tahir

**Affiliations:** a Institute of Chemical Sciences, University of Peshawar, Peshawar, Pakistan; b Department of Physics, University of Sargodha, Sargodha, Pakistan

## Abstract

In the title salt, C_7_H_8_NO_4_^+^·Cl^−^, the organic group is planar with an r.m.s. deviation of 0.0265 Å. An *S*(6) ring motif is formed due to an intra­molecular O—H⋯O hydrogen bond. The compound consists of dimers due to inter­molecular O—H⋯O hydrogen bonds with an *R*_2_^2^(8) ring motif. The dimers are inter­linked through strong N—H⋯Cl and O—H⋯Cl hydrogen bonds, resulting in a three-dimensional polymeric network.

## Related literature

For related structures, see: Bendjeddou *et al.* (2009 ▶); Dobson & Gerkin (1998 ▶). For graph-set notation, see: Bernstein *et al.* (1995 ▶).
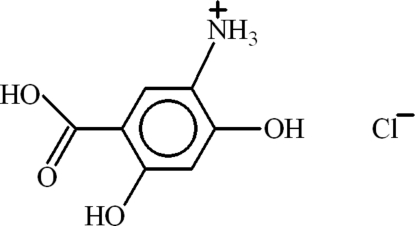


## Experimental

### 

#### Crystal data

C_7_H_8_NO_4_^+^·Cl^−^
*M* *_r_* = 205.59
Monoclinic, 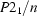

*a* = 5.0667 (3) Å
*b* = 28.4071 (13) Å
*c* = 6.3966 (3) Å
β = 97.649 (3)°
*V* = 912.47 (8) Å^3^
*Z* = 4
Mo *K*α radiation
μ = 0.40 mm^−1^
*T* = 296 K
0.28 × 0.18 × 0.16 mm

#### Data collection

Bruker Kappa APEXII CCD diffractometer
Absorption correction: multi-scan (*SADABS*; Bruker, 2005 ▶) *T*_min_ = 0.926, *T*_max_ = 0.935
7094 measured reflections
1635 independent reflections
1108 reflections with *I* > 2σ(*I*)
*R*_int_ = 0.052

#### Refinement

*R*[*F* ^2^ > 2σ(*F* ^2^)] = 0.044
*wR*(*F* ^2^) = 0.098
*S* = 1.01
1635 reflections
128 parameters
H atoms treated by a mixture of independent and constrained refinement
Δρ_max_ = 0.23 e Å^−3^
Δρ_min_ = −0.22 e Å^−3^

### 

Data collection: *APEX2* (Bruker, 2009 ▶); cell refinement: *SAINT* (Bruker, 2009 ▶); data reduction: *SAINT*; program(s) used to solve structure: *SHELXS97* (Sheldrick, 2008 ▶); program(s) used to refine structure: *SHELXL97* (Sheldrick, 2008 ▶); molecular graphics: *ORTEP-3 for Windows* (Farrugia, 1997 ▶) and *PLATON* (Spek, 2009 ▶); software used to prepare material for publication: *WinGX* (Farrugia, 1999 ▶) and *PLATON*.

## Supplementary Material

Crystal structure: contains datablocks global, I. DOI: 10.1107/S1600536810033337/si2289sup1.cif

Structure factors: contains datablocks I. DOI: 10.1107/S1600536810033337/si2289Isup2.hkl

Additional supplementary materials:  crystallographic information; 3D view; checkCIF report

## Figures and Tables

**Table 1:** Hydrogen-bond geometry (Å, °)

*D*—H⋯*A*	*D*—H	H⋯*A*	*D*⋯*A*	*D*—H⋯*A*
O1—H1⋯O2^i^	0.85 (3)	1.81 (3)	2.653 (3)	177 (4)
N1—H1*A*⋯Cl1^ii^	0.89	2.24	3.124 (3)	176
N1—H1*B*⋯Cl1^iii^	0.89	2.29	3.176 (3)	173
N1—H1*C*⋯Cl1^iv^	0.89	2.35	3.217 (2)	163
O3—H3⋯O2	0.85 (4)	1.84 (3)	2.632 (3)	154 (3)
O4—H4*A*⋯Cl1^v^	0.85 (3)	2.15 (3)	2.985 (2)	170 (3)

Symmetry codes: (i) 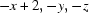; (ii) 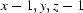; (iii) 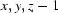; (iv) 
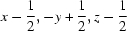
; (v) 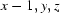.

**Fig. 1.**
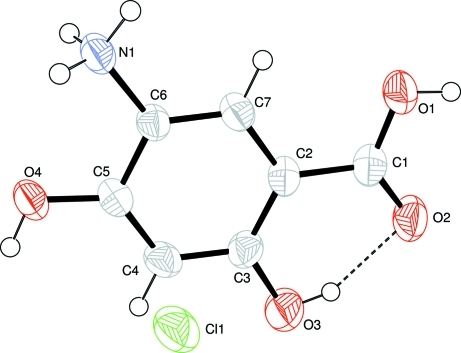
View of the title compound with the atom numbering scheme. The displacement ellipsoids are drawn at the 50% probability level. H-atoms are shown as small spheres of arbitrary radii. The dotted line represents the intramolecular H-bonding.

**Fig. 2.**
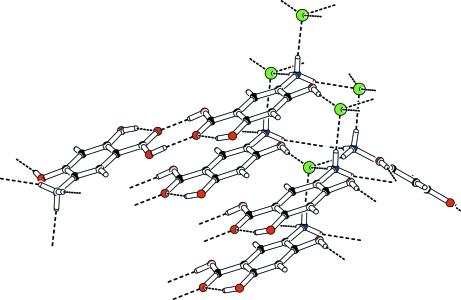
The partial packing (PLATON; Spek, 2009) which shows that molecules form dimers which are interlinked through H-bondings to form a three-dimensional polymeric network.

## supplementary crystallographic information

### Comment

The title compound (I, Fig. 1) has been prepared for derivatization and for the synthesis of metallic complexes.

The crystal structure of (II) *i.e.*, 5-ammoniosalicylic Acid chloride monohydrate (Dobson & Gerkin, 1998) and (III) *i.e.*, bis(3-carboxyanilinum) bis(perchlorate) monohydrate (Bendjeddou *et al.*, 2009) have been published which are related to the title compound.

In (I), the organic group (C1—C7/O1—O4/N1) is planar with r. m. s. deviation of 0.0265 Å. There exist a strong intramolecular H-bond of O—H···O type (Table 1, Fig. 1) completing an S(6) ring motif (Bernstein *et al.*, 1995) in the organic part. The title compound consists of dimers due to intermolecular H-bond of O—H···O type (Table 1, Fig. 2) completing *R*_2_^2^(8) ring motif. The dimers are interlinked through strong H-bondings of N—H···Cl and O—H···Cl types (Table 1, Fig. 2) resulting in a three dimensional polymeric network.

### Experimental

Concentrated nitric acid (2 mL, 67%) was added drop by drop to β-resorcylic acid (1 g, 97%, 6.3 mmol) in a round bottom flask. The mixture was protected from moisture by CaCl_2_ (anhydrous) tube and was allowed to stand for 12 h at room temperature. Then the reaction mixture was diluted with water. The crude material was filtered and recrystallized from water to affoard the 5-nitro-β-resorcylic acid.

Then a mixture of 5-nitro-β-resorcylic acid (1.5 g, 7.5 mmol), tin (3 g, 25 mmol), concentrated hydrochloric acid (8 ml, 37%) and absolute ethanol (5 ml) were taken in a 100 ml round bottom flask and heated under reflux with stirring for 40 min. The completion of the reaction was monitored by TLC. The reaction mixture was filtered to remove any unreacted tin. The filtrate was kept for seven days to afford light green prisms of (I).

### Refinement

The coordinates of H-atoms of hydroxy groups were refined. The H-atoms were positioned geometrically (N—H = 0.89, C–H = 0.93 Å) and refined as riding with *U*_iso_(H) = x*U*_eq_(C, N, O), where x = 1.2 for all H-atoms.

### Crystal data

**Table:**

C_7_H_8_NO_4_^+^·Cl^−^	*F*(000) = 424
*M**_r_* = 205.59	*D*_x_ = 1.497 Mg m^−^^3^
Monoclinic, *P*2_1_/*n*	Mo *K*α radiation, λ = 0.71073 Å
Hall symbol: -P 2yn	Cell parameters from 1108 reflections
*a* = 5.0667 (3) Å	θ = 2.9–25.2°
*b* = 28.4071 (13) Å	µ = 0.40 mm^−^^1^
*c* = 6.3966 (3) Å	*T* = 296 K
β = 97.649 (3)°	Prism, light green
*V* = 912.47 (8) Å^3^	0.28 × 0.18 × 0.16 mm
*Z* = 4	

### Data collection

**Table:**

Bruker Kappa APEXII CCD diffractometer	1635 independent reflections
Radiation source: fine-focus sealed tube	1108 reflections with *I* > 2σ(*I*)
graphite	*R*_int_ = 0.052
Detector resolution: 8.10 pixels mm^-1^	θ_max_ = 25.2°, θ_min_ = 2.9°
ω scans	*h* = −5→6
Absorption correction: multi-scan (*SADABS*; Bruker, 2005)	*k* = −34→32
*T*_min_ = 0.926, *T*_max_ = 0.935	*l* = −7→7
7094 measured reflections	

### Refinement

**Table:**

Refinement on *F*^2^	Primary atom site location: structure-invariant direct methods
Least-squares matrix: full	Secondary atom site location: difference Fourier map
*R*[*F*^2^ > 2σ(*F*^2^)] = 0.044	Hydrogen site location: inferred from neighbouring sites
*wR*(*F*^2^) = 0.098	H atoms treated by a mixture of independent and constrained refinement
*S* = 1.01	*w* = 1/[σ^2^(*F*_o_^2^) + (0.036*P*)^2^ + 0.4226*P*] where *P* = (*F*_o_^2^ + 2*F*_c_^2^)/3
1635 reflections	(Δ/σ)_max_ < 0.001
128 parameters	Δρ_max_ = 0.23 e Å^−^^3^
0 restraints	Δρ_min_ = −0.22 e Å^−^^3^

### Special details

**Table:**

Geometry. Bond distances, angles *etc*. have been calculated using the rounded fractional coordinates. All su's are estimated from the variances of the (full) variance-covariance matrix. The cell e.s.d.'s are taken into account in the estimation of distances, angles and torsion angles
Refinement. Refinement of *F*^2^ against ALL reflections. The weighted *R*-factor *wR* and goodness of fit *S* are based on *F*^2^, conventional *R*-factors *R* are based on *F*, with *F* set to zero for negative *F*^2^. The threshold expression of *F*^2^ > σ(*F*^2^) is used only for calculating *R*-factors(gt) *etc*. and is not relevant to the choice of reflections for refinement. *R*-factors based on *F*^2^ are statistically about twice as large as those based on *F*, and *R*- factors based on ALL data will be even larger.

### Fractional atomic coordinates and isotropic or equivalent isotropic displacement parameters (Å^2^)

**Table:**

	*x*	*y*	*z*	*U*_iso_*/*U*_eq_	
O1	0.8567 (5)	0.05383 (8)	−0.1244 (4)	0.0527 (8)	
O2	0.7828 (4)	0.01248 (8)	0.1583 (3)	0.0553 (8)	
O3	0.4286 (5)	0.04378 (8)	0.3905 (4)	0.0620 (10)	
O4	0.0113 (4)	0.18833 (7)	0.1726 (3)	0.0498 (8)	
N1	0.2950 (5)	0.19898 (8)	−0.1420 (4)	0.0404 (8)	
C1	0.7367 (6)	0.04694 (11)	0.0429 (5)	0.0420 (11)	
C2	0.5471 (5)	0.08321 (10)	0.0823 (4)	0.0373 (10)	
C3	0.4020 (6)	0.08012 (10)	0.2540 (5)	0.0404 (10)	
C4	0.2197 (6)	0.11460 (10)	0.2882 (5)	0.0415 (11)	
C5	0.1841 (6)	0.15295 (10)	0.1561 (4)	0.0366 (10)	
C6	0.3332 (6)	0.15690 (10)	−0.0114 (4)	0.0343 (9)	
C7	0.5084 (6)	0.12258 (10)	−0.0495 (4)	0.0376 (10)	
Cl1	0.75184 (16)	0.19834 (3)	0.56278 (12)	0.0476 (3)	
H1	0.974 (7)	0.0328 (12)	−0.131 (5)	0.0632*	
H1A	0.13993	0.19710	−0.22519	0.0485*	
H1B	0.42649	0.20130	−0.22080	0.0485*	
H1C	0.29515	0.22425	−0.05970	0.0485*	
H3	0.543 (7)	0.0264 (13)	0.342 (5)	0.0743*	
H4	0.12136	0.11186	0.40041	0.0498*	
H4A	−0.063 (6)	0.1874 (11)	0.284 (5)	0.0597*	
H7	0.60291	0.12534	−0.16402	0.0451*	

### Atomic displacement parameters (Å^2^)

**Table:**

	*U*^11^	*U*^22^	*U*^33^	*U*^12^	*U*^13^	*U*^23^
O1	0.0541 (15)	0.0463 (15)	0.0613 (14)	0.0190 (11)	0.0212 (13)	0.0073 (11)
O2	0.0518 (14)	0.0443 (14)	0.0728 (15)	0.0160 (12)	0.0189 (12)	0.0144 (12)
O3	0.0706 (18)	0.0536 (16)	0.0664 (16)	0.0192 (13)	0.0267 (14)	0.0218 (13)
O4	0.0599 (15)	0.0434 (14)	0.0525 (13)	0.0153 (12)	0.0311 (12)	0.0053 (10)
N1	0.0393 (14)	0.0373 (15)	0.0483 (14)	0.0049 (12)	0.0195 (12)	0.0009 (12)
C1	0.0349 (18)	0.0375 (18)	0.0538 (19)	0.0028 (15)	0.0067 (16)	0.0026 (16)
C2	0.0321 (16)	0.0322 (17)	0.0480 (18)	0.0030 (14)	0.0073 (14)	0.0010 (14)
C3	0.0410 (18)	0.0357 (18)	0.0453 (18)	0.0028 (15)	0.0085 (15)	0.0084 (14)
C4	0.0448 (19)	0.0414 (19)	0.0415 (17)	−0.0006 (16)	0.0179 (15)	0.0034 (15)
C5	0.0361 (18)	0.0348 (18)	0.0406 (16)	−0.0005 (14)	0.0116 (14)	−0.0047 (14)
C6	0.0343 (16)	0.0297 (17)	0.0409 (16)	0.0009 (13)	0.0125 (14)	0.0028 (13)
C7	0.0346 (17)	0.0381 (18)	0.0417 (16)	0.0014 (14)	0.0115 (14)	−0.0018 (14)
Cl1	0.0500 (5)	0.0494 (5)	0.0480 (4)	0.0043 (4)	0.0240 (4)	0.0048 (4)

### Geometric parameters (Å, °)

**Table:**

O1—C1	1.314 (4)	N1—H1C	0.8900
O2—C1	1.230 (4)	C1—C2	1.453 (4)
O3—C3	1.347 (4)	C2—C7	1.399 (4)
O4—C5	1.346 (4)	C2—C3	1.403 (4)
O1—H1	0.85 (3)	C3—C4	1.384 (4)
O3—H3	0.85 (4)	C4—C5	1.376 (4)
O4—H4A	0.85 (3)	C5—C6	1.395 (4)
N1—C6	1.456 (4)	C6—C7	1.362 (4)
N1—H1A	0.8900	C4—H4	0.9300
N1—H1B	0.8900	C7—H7	0.9300
			
Cl1···N1^i^	3.176 (3)	C1···C1^vi^	3.580 (4)
Cl1···N1^ii^	3.124 (3)	C1···O2^vi^	3.245 (4)
Cl1···C7^i^	3.622 (3)	C1···O2^v^	3.357 (4)
Cl1···O4^iii^	2.985 (2)	C1···C4^iii^	3.335 (4)
Cl1···N1^iv^	3.217 (2)	C2···C4^iii^	3.598 (4)
Cl1···H4A^iii^	2.15 (3)	C3···C1^viii^	3.587 (4)
Cl1···H1A^ii^	2.2400	C4···C2^viii^	3.598 (4)
Cl1···H1B^i^	2.2900	C4···C1^viii^	3.335 (4)
Cl1···H1C^iv^	2.3500	C7···O4^iii^	3.322 (4)
Cl1···H7^i^	2.8800	C7···Cl1^ix^	3.622 (3)
O1···O2^v^	2.653 (3)	C1···H1^v^	2.72 (3)
O2···O3	2.632 (3)	C1···H3	2.34 (3)
O2···C1^vi^	3.245 (4)	H1···O2^v^	1.81 (3)
O2···O1^v^	2.653 (3)	H1···C1^v^	2.72 (3)
O2···C1^v^	3.357 (4)	H1···H1^v^	2.50 (5)
O3···O3^vii^	2.899 (3)	H1A···Cl1^x^	2.2400
O3···O2	2.632 (3)	H1A···O4	2.7200
O4···N1	2.642 (3)	H1B···H7	2.3500
O4···Cl1^viii^	2.985 (2)	H1B···Cl1^ix^	2.2900
O4···C7^viii^	3.322 (4)	H1C···O4	2.4300
O1···H7	2.4000	H1C···Cl1^xi^	2.3500
O2···H3	1.84 (3)	H3···C1	2.34 (3)
O2···H1^v^	1.81 (3)	H3···O2	1.84 (3)
O3···H3^vii^	2.62 (3)	H3···H3^vii^	2.60 (5)
O4···H1C	2.4300	H3···O3^vii^	2.62 (3)
O4···H1A	2.7200	H4···H4A	2.4200
N1···Cl1^ix^	3.176 (3)	H4A···H4	2.4200
N1···Cl1^x^	3.124 (3)	H4A···Cl1^viii^	2.15 (3)
N1···O4	2.642 (3)	H7···Cl1^ix^	2.8800
N1···Cl1^xi^	3.217 (2)	H7···O1	2.4000
C1···C3^iii^	3.587 (4)	H7···H1B	2.3500
			
C1—O1—H1	110 (2)	O3—C3—C4	116.8 (3)
C3—O3—H3	103 (2)	C2—C3—C4	120.7 (3)
C5—O4—H4A	114 (2)	O3—C3—C2	122.5 (3)
C6—N1—H1C	109.00	C3—C4—C5	120.0 (3)
H1A—N1—H1B	109.00	C4—C5—C6	119.7 (3)
C6—N1—H1A	109.00	O4—C5—C6	115.1 (2)
C6—N1—H1B	109.00	O4—C5—C4	125.2 (3)
H1B—N1—H1C	109.00	N1—C6—C5	117.5 (3)
H1A—N1—H1C	109.00	N1—C6—C7	121.7 (2)
O1—C1—C2	115.1 (3)	C5—C6—C7	120.8 (3)
O1—C1—O2	122.4 (3)	C2—C7—C6	120.5 (3)
O2—C1—C2	122.5 (3)	C3—C4—H4	120.00
C1—C2—C7	120.4 (2)	C5—C4—H4	120.00
C3—C2—C7	118.4 (3)	C2—C7—H7	120.00
C1—C2—C3	121.2 (3)	C6—C7—H7	120.00
			
O1—C1—C2—C3	179.2 (3)	O3—C3—C4—C5	179.4 (3)
O1—C1—C2—C7	−1.8 (4)	C2—C3—C4—C5	−1.6 (5)
O2—C1—C2—C3	−1.2 (5)	C3—C4—C5—O4	179.3 (3)
O2—C1—C2—C7	177.8 (3)	C3—C4—C5—C6	−0.2 (4)
C1—C2—C3—O3	−0.2 (4)	O4—C5—C6—N1	2.6 (4)
C1—C2—C3—C4	−179.2 (3)	O4—C5—C6—C7	−177.7 (3)
C7—C2—C3—O3	−179.2 (3)	C4—C5—C6—N1	−177.8 (3)
C7—C2—C3—C4	1.8 (4)	C4—C5—C6—C7	1.9 (4)
C1—C2—C7—C6	−179.2 (3)	N1—C6—C7—C2	178.0 (3)
C3—C2—C7—C6	−0.2 (4)	C5—C6—C7—C2	−1.7 (4)

Symmetry codes: (i) *x*, *y*, *z*+1; (ii) *x*+1, *y*, *z*+1; (iii) *x*+1, *y*, *z*; (iv) *x*+1/2, −*y*+1/2, *z*+1/2; (v) −*x*+2, −*y*, −*z*; (vi) −*x*+1, −*y*, −*z*; (vii) −*x*+1, −*y*, −*z*+1; (viii) *x*−1, *y*, *z*; (ix) *x*, *y*, *z*−1; (x) *x*−1, *y*, *z*−1; (xi) *x*−1/2, −*y*+1/2, *z*−1/2.

### Hydrogen-bond geometry (Å, °)

**Table:**

*D*—H···*A*	*D*—H	H···*A*	*D*···*A*	*D*—H···*A*
O1—H1···O2^v^	0.85 (3)	1.81 (3)	2.653 (3)	177 (4)
N1—H1A···Cl1^x^	0.89	2.24	3.124 (3)	176
N1—H1B···Cl1^ix^	0.89	2.29	3.176 (3)	173
N1—H1C···Cl1^xi^	0.89	2.35	3.217 (2)	163
O3—H3···O2	0.85 (4)	1.84 (3)	2.632 (3)	154 (3)
O4—H4A···Cl1^viii^	0.85 (3)	2.15 (3)	2.985 (2)	170 (3)

Symmetry codes: (v) −*x*+2, −*y*, −*z*; (x) *x*−1, *y*, *z*−1; (ix) *x*, *y*, *z*−1; (xi) *x*−1/2, −*y*+1/2, *z*−1/2; (viii) *x*−1, *y*, *z*.
